# Preoperative Abdominal Aortic Aneurysm Diameter Is Associated with Long-Term Durability After Endovascular Aortic Aneurysm Repair: A Multicenter Real-World Italian Cohort Study

**DOI:** 10.3390/jcdd13070325

**Published:** 2026-07-12

**Authors:** Giulio Accarino, Davide Esposito, Raffaele Serra, Raffaele Pulli, Sara Speziali, Nicola Troisi, Raffaella Berchiolli, Giovanni Pratesi, Enrico Cappello, Giancarlo Accarino, Jean-Paul P. M. de Vries, Umberto Marcello Bracale

**Affiliations:** 1 Vascular Surgery Unit, Department of Public Health, University Federico II of Naples, 80138 Naples, Italy; g.accarino@live.it (G.A.); umbertomarcello.bracale@unina.it (U.M.B.); 2 Department of Medicine, Surgery and Dentistry, University of Salerno, 84084 Salerno, Italy; giancarloaccarin@me.com; 3 Department of Surgery, Division of Vascular Surgery, University Medical Centre Groningen, University of Groningen, 9713 GZ Groningen, The Netherlands; j.p.p.m.de.vries@umcg.nl; 4 Department of Surgical and Integrated Diagnostic Sciences, University of Genoa, 16132 Genoa, Italy; davide.esposito@unige.it (D.E.); giovanni.pratesi@unige.it (G.P.); 5 Clinic of Vascular and Endovascular Surgery, IRCCS Ospedale Policlinico San Martino, 16132 Genoa, Italy; 6 Interuniversity Center of Phlebolymphology (CIFL), International Research and Educational Program in Clinical and Experimental Biotechnology, University ‘‘Magna Graecia’’ of Catanzaro, 88100 Catanzaro, Italy; 7 Department of Medical and Surgical Sciences, University ‘‘Magna Graecia’’ of Catanzaro, 88100 Catanzaro, Italy; 8 Vascular Surgery Department, Careggi University Hospital, University of Florence, 16132 Florence, Italy; raffaele.pulli@unifi.it (R.P.); sara.speziali@unifi.it (S.S.); 9 Vascular Surgery Unit, Department of Translational Research and New Technologies in Medicine and Surgery, University of Pisa, 56126 Pisa, Italy; nicola.troisi@unipi.it (N.T.); raffaella.berchiolli@unipi.it (R.B.); 10 Unit of Vascular and Endovascular Surgery, IRCCS Neuromed, 86077 Pozzilli, Italy; enrico.cappello@yahoo.it

**Keywords:** endovascular aneurysm repair, abdominal aortic aneurysm, aneurysm diameter, endoleak, reintervention

## Abstract

**Objective:** To assess whether baseline abdominal aortic aneurysm (AAA) diameter is associated with long-term durability after elective EVAR, after accounting for relevant anatomic differences. **Methods:** Consecutive patients undergoing elective EVAR for infrarenal fusiform AAA between 2011 and 2022 at four Italian centers were analyzed. Ruptured, saccular, and adjunctive proximal or distal procedures were excluded. Patients were stratified as AAA ≤ 50 mm or >50 mm. Outcomes included any endoleak, reintervention, aneurysm-related death, sac behavior, and all-cause mortality. Analyses used cumulative incidence functions, Fine–Gray competing-risk models, multivariable Cox regression, and propensity-score matching incorporating key anatomic variables. **Results:** Among 1505 patients, 499 (33.2%) had AAA ≤ 50 mm. Median follow-up was 49 months. Larger aneurysms showed higher 10-year cumulative incidence of any endoleak (33.9% vs. 15.8%, Gray *p* < 0.001), type 1a endoleak (16.5% vs. 6.1%, Gray *p* < 0.001), reintervention (24.7% vs. 16.2%, Gray *p* < 0.001), and aneurysm-related death (5.4% vs. 2.4%, Gray *p* = 0.028). In anatomy-adjusted multivariable Cox models, each 1 cm increase in diameter was associated with higher hazards of any endoleak (HR 1.22, 95% CI 1.11–1.34; *p* < 0.001), type 2 endoleak (HR 1.26, 95% CI 1.12–1.43; *p* < 0.001), reintervention (HR 1.19, 95% CI 1.07–1.32; *p* = 0.001), and aneurysm-related mortality (HR 1.46, 95% CI 1.19–1.81; *p* < 0.001). In propensity-score-matched analyses, AAA ≤ 50 mm was associated with lower hazards of any endoleak, HR 0.39, 95% CI 0.28–0.55; *p* < 0.001, and reintervention, HR 0.40, 95% CI 0.26–0.62; *p* < 0.001. **Conclusions:** Baseline AAA diameter adds prognostic information beyond standard anatomic features and may help tailor post-EVAR surveillance. **What is new?:** This multicenter real-world study shows that baseline abdominal aortic aneurysm diameter provides continuous prognostic information of clinical outcomes after elective EVAR. Larger preoperative diameter was associated with higher long-term risk of endoleak and reintervention, supporting the use of baseline sac size as one component of individualized post-EVAR surveillance planning.

## 1. Introduction

Abdominal aortic aneurysm (AAA) remains a major cause of morbidity and mortality in older adults [1]. Despite early detection and surveillance campaigns, there is still a significant burden for this disease [2]. Contemporary guidelines recommend surveillance for most asymptomatic aneurysms below the conventional intervention threshold (50 mm in women and 55 mm in men) [3,4]. The evidence supporting surveillance of small asymptomatic AAAs is largely based on randomized trials comparing early repair with continued surveillance. In the UK Small Aneurysm Trial [5], patients with 4.0–5.5 cm AAAs were randomized to early open repair or ultrasound surveillance, with repair recommended after threshold enlargement, rapid expansion, or symptom development. After a mean follow-up of 4.6 years, early open repair did not reduce all-cause mortality. Moreover, extended follow-up to 12 years confirmed no long-term survival advantage of early open repair [6]. Similarly, the ADAM trial [7] randomized patients with 4.0–5.4 cm AAAs to immediate open repair or surveillance and found no survival benefit from open repair during follow-up. These trials provide the clinical background for surveillance thresholds, but they did not evaluate whether the preoperative diameter remains prognostically relevant to post-EVAR outcomes.

Some authors have speculated that contemporary improvements in cardiovascular prevention and the best medical therapy may have attenuated rupture risk compared with the era of UKSAT and ADAM [7]. However, no high-quality evidence currently demonstrates a measurable reduction in AAA rupture attributable to medical therapy alone, and this assumption remains unproven [8]. In contemporary practice, a substantial proportion of elective AAA repairs are performed below these conventional thresholds [9,10,11]. Recent analyses from large registries, including VQI and multicenter academic cohorts, report that 30–40% of EVAR procedures occur in aneurysms < 55 mm in men and ≤50 mm in women [9,10,11]. Repair below conventional diameter thresholds is therefore observed in contemporary vascular practice, although its appropriateness depends on patient-level clinical and anatomical factors that are not captured by diameter alone. Anatomical progression during surveillance may erode standard EVAR suitability. The proximal aortic neck—the segment between the renal arteries and the aneurysm sac—is critical for fixation. Contemporary EVAR devices and adjuncts may mitigate these risks. In a recently published cohort, each millimeter of sealing zone length conferred an 8–12% reduction in type 1a endoleak hazard [12]. This further amplifies the importance of a non-hostile anatomy when using standard EVAR. Improvements in endograft design, operator experience and peri-operative care and best medical treatment have lowered mortality and morbidity [10,11,12]. Whether the baseline diameter conveys independent prognostic information for EVAR durability in real-world practice remains unclear. This study examines diameter as an independent and continuous predictor of post-EVAR outcomes in a large multicenter cohort.

## 2. Materials and Methods

### 2.1. Study Design

This is a retrospective, multicenter study that includes all consecutive AAA patients undergoing EVAR from November 2011 to November 2022 in four centers.

The inclusion criteria were infrarenal fusiform degenerative AAA treated by elective EVAR. Ruptured AAA and urgent/emergent EVAR were excluded. Symptomatic but unruptured AAAs were not a formal exclusion criterion if the procedure was performed electively.

Exclusion criteria included:

- EVAR procedures performed for different reasons than degenerative AAA (penetrating aortic ulcers and dissections);

- EVAR performed for saccular aneurysms (focal, asymmetric dilation of the abdominal aorta);

- Urgent/emergent EVAR for ruptured aneurysms;

- EVAR which required adjunctive proximal and/or distal procedures such as EndoAnchor placement, Chimney stent grafts, fenestrated/branched stent grafts, and iliac branch devices;

- EVAR patients with unavailable follow-up.

Demographics, clinical, anatomical, periprocedural, and in-hospital data were systematically collected for each patient. Neck thrombus and calcium patterns were summarized as favorable (absent/isolated) versus unfavorable (semi circumferential/circumferential) to provide a concise anatomical descriptor. Definitions conformed to reporting standards [13].

For descriptive comparisons, patients were stratified a priori by the maximum preoperative AAA diameter at the conventional 50 mm threshold (chosen to represent sub-threshold repair in women and the lower bound of threshold repair in men, and for comparability with prior registry analyses). This dichotomization was used for descriptive tables and unadjusted survival curves; it was not intended to represent a data-derived optimal cut-off. Primary inference was performed on diameter as a continuous variable [3,4]. Indications for elective EVAR in contemporary practice may include clinical and anatomical factors beyond maximum aortic diameter, including sex-specific thresholds, rapid growth, symptoms, concomitant iliac aneurysm, patient preference, and multidisciplinary judgment.

Treatment decisions were made independently at each participating center in routine multidisciplinary vascular practice and were not protocolized for this retrospective study. Individual indications for repair below conventional diameter thresholds were not uniformly recorded; accordingly, this study was not designed to assess guideline adherence or treatment appropriateness, but rather outcomes among patients already selected for elective EVAR [3,4].

All patients gave written consent for the anonymous collection of clinical data on the standard consent form provided by each institute. This study was approved by the ethical committee “Campania 2” (n. 2024/21860).

The checklist of items conformed to the guidelines of the Strengthening the Reporting of Observational Studies in Epidemiology (STROBE) statement [14].

### 2.2. Follow-Up Algorithms and Study Outcomes

Follow-up protocols included a post-operative computed tomography angiography (CTA) within one month of the index procedure. A one-year CTA follow-up control was conducted at each institution. Subsequent follow-up assessments were performed at the discretion of individual participating centers, typically involving duplex ultrasound or CTA at least once every year. AAA maximum diameter measurements at follow-up were compared with pre-operative dimensions to allow sac behavior stratification. Additional follow-up data were obtained through in-person visits or supplemented by telephone interviews, medical record reviews, and consultation of regional electronic mortality registries.

Primary outcomes were time to first occurrence of any endoleak, reintervention, and aneurysm-related death. Secondary outcomes included sac behavior and overall mortality.

### 2.3. Statistical Analysis

Continuous variables were summarized as the median and interquartile range and compared between the ≤50 mm and >50 mm groups using Mann–Whitney U tests. Categorical variables were summarized as n/N (%) and compared using Pearson χ^2^ tests; Fisher’s exact test was used when the expected cell counts were <5. Variables used to define the diameter groups, including the maximum baseline AAA diameter, were not formally compared. Primary inference was based on time-to-event analyses, with the baseline AAA diameter modeled as a continuous variable per 1 cm increase. Cumulative incidence functions were estimated for each diameter group and compared using Gray’s test. For endoleak and reintervention endpoints, all-cause death without prior event was treated as the competing event. For aneurysm-related mortality, non-aneurysm-related death was treated as the competing event. Fine–Gray competing-risk models were used to estimate subdistribution hazard ratios for the baseline aneurysm diameter. Missing anatomical covariates were handled using multiple imputation by chained equations with 50 imputations. Predictive mean matching was used for continuous variables. Patterns of missingness for variables used in multivariable and matched analyses are reported in Supplementary Table S1. Outcome indicators, event times, center, sex, baseline aneurysm diameter, and device type were not imputed. Cox proportional hazards models were fitted within each imputed dataset and pooled using Rubin’s rules.

Multivariable Cox models were specified according to the endpoint frequency and anatomical relevance. Common endpoints were evaluated using core anatomical models including age, sex, proximal neck length, proximal neck diameter, iliac diameters, and patent lumbar arteries. Type Ib endoleak and aneurysm-related mortality were evaluated using reduced anatomical models including age, sex, proximal neck length, and proximal neck diameter to avoid overfitting. Additional sensitivity analyses accounted for the center using fixed-effect and stratified Cox models, and for the device group in patients with available device information.

Propensity-score matching was performed as an exploratory sensitivity analysis across the 50 imputed datasets using 1:1 nearest-neighbor matching without replacement, based on a logistic-regression propensity score. The propensity model included proximal neck length, proximal neck diameter, infrarenal neck angulation, right and left common iliac artery diameters, patent lumbar arteries, age, and sex. The covariate balance was summarized using standardized mean differences. Outcome associations in the matched cohorts were estimated using Cox models with robust standard errors clustered by matched subclass and pooled across imputations using Rubin’s rules.

The incremental prognostic value of the baseline diameter was explored for endpoints with at least 100 events by comparing core anatomical Cox models with and without the diameter, reporting changes in apparent Harrell’s C-index and Akaike information criterion across imputed datasets. No conventional derived cut-off was estimated because the primary endpoints were censored time-to-event outcomes, several with competing mortality, and because the study was not designed to identify a new treatment threshold.

All tests were two-sided, with statistical significance set at *p* < 0.05.

All analyses were performed using JASP version 0.95.4 (JASP Team, Amsterdam, The Netherlands) and R (version 4.5.2 using the dplyr, mice, survival, cmprsk, MatchIt, and ggplot2 packages).

## 3. Results

### 3.1. Study Cohort and Baseline Characteristics

A total of 1505 patients were included; 499 patients (33.2%) presented with a maximum aneurysm diameter ≤ 50 mm (small-AAA group). AAA ≤ 50 mm was associated with younger age (73 versus 75 years, *p* < 0.001), longer (23 versus 20 mm, *p* < 0.001) and narrower proximal necks (23 versus 25 mm, *p* < 0.001), lower neck angulation 20 versus 27, *p* < 0.001), and smaller iliac diameters. Diabetes was more frequent in the AAA ≤ 50 mm group, 24.4% versus 16.7%; *p* < 0.001, as was dyslipidemia, 61.1% versus 54.3%; *p* = 0.023 (Table 1).

Median oversizing of the main body did not differ between groups; patterns of neck thrombus and calcium were similar across diameter strata. The proportion of EVARs performed outside device instructions for use (IFU) was comparable in the two groups (10.8% vs. 12.8%; *p* = 0.348).

### 3.2. Perioperative and Long-Term Outcomes

The median follow-up time was 49 (20–78) months. During follow-up, 290 endoleaks and 188 reinterventions were reported in the whole cohort. Any endoleak occurred less frequently after EVAR for AAA ≤ 50 mm, 11.1% versus 23.7%; *p* < 0.001. This difference was mainly driven by lower rates of type 1a endoleak, 2.8% versus 7.2%; *p* < 0.001, and type 2 endoleak, 7.0% versus 14.8%; *p* < 0.001. Type Ib and type 3 endoleaks did not differ significantly between groups. Table 2 summarizes crude perioperative and follow-up event counts.

Median follow-up was longer in the AAA ≤ 50 mm group, 55 months, IQR 23–84, than in the AAA > 50 mm group, 43 months, IQR 16–74; *p* < 0.001.

Endurant (Medtronic Inc., Minneapolis, MN, USA) represented the most employed stent-graft overall (51%), with AFX (Endologix LLC, Irvine, CA, USA) accounting for 18%. Device use differed across diameter strata: AFX was more frequently used in aneurysms ≤50 mm (38% vs. 9%), whereas Endurant predominated in larger aneurysms.

### 3.3. Cumulative Incidence Functions

**Figure 1:**
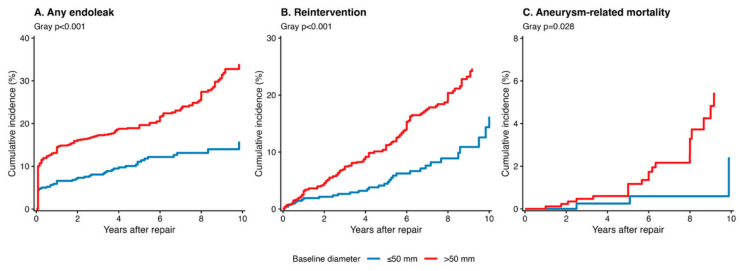
Cumulative incidence functions for main post-repair durability endpoints after elective endovascular abdominal aortic aneurysm repair, stratified by baseline aneurysm diameter. (**A**) Any endoleak, (**B**) reintervention, and (**C**) aneurysm-related mortality. For endoleak and reintervention endpoints, all-cause death without prior event was treated as the competing event. For aneurysm-related mortality, non-aneurysm-related death was treated as the competing event. Gray *p* values compare cumulative incidence functions between aneurysms ≤50 mm and >50 mm.

Cumulative incidence functions for the main durability endpoints are shown in Figure 1.

Cumulative incidence analyses are shown in Table 3. At 10 years, the cumulative incidence of any endoleak was 15.8% in the AAA ≤ 50 mm group and 33.9% in the AAA > 50 mm group; Gray *p* < 0.001. Similar differences were observed for type 1a endoleak, 6.1% versus 16.5%; Gray *p* = 0.001, and type 2 endoleak, 7.9% versus 18.2%; Gray *p* < 0.001. The 10-year cumulative incidence of reintervention was 16.2% in the AAA ≤ 50 mm group and 24.7% in the AAA > 50 mm group; Gray *p* < 0.001. Aneurysm-related mortality remained infrequent in both groups but was higher in patients with AAA >50 mm, 5.4% versus 2.4% at 10 years; Gray *p* = 0.028. Type Ib endoleak did not differ significantly between groups. Aneurysm-related death was uncommon in both groups during early follow-up, and the cumulative incidence curves remained close for approximately 5 years. Separation became more evident during longer-term follow-up, with 10-year cumulative incidence estimates of 2.4% in the AAA ≤ 50 mm group and 5.4% in the AAA > 50 mm group; Gray *p* = 0.028.

Non-parametric cumulative incidence functions were estimated using endpoint-specific time-to-event definitions. For endoleak and reintervention endpoints, all-cause death without prior event was treated as the competing event. For aneurysm-related mortality, non-aneurysm-related death was treated as the competing event. Values are rounded to one decimal.

### 3.4. Multivariable Cox and Fine–Gray Models

In endpoint-adapted multivariable models, baseline aneurysm diameter was modeled continuously per 1 cm increase and reported in Table 4.

Per 1 cm increase, the baseline diameter was associated with any endoleak, Cox HR 1.22, 95% CI 1.11–1.34; *p* < 0.001, and Fine–Gray sHR 1.20, 95% CI 1.10–1.31; *p* < 0.001. The baseline diameter was also associated with reintervention, Cox HR 1.19, 95% CI 1.07–1.32; *p* = 0.001, and Fine–Gray sHR 1.15, 95% CI 1.05–1.27; *p* = 0.004. In adjusted models, the baseline diameter remained associated with both type 1a and type 2 endoleaks. Per 1 cm increase in the baseline diameter, type 1a endoleak showed a Cox HR of 1.21, 95% CI 1.06–1.38; *p* = 0.006, and a Fine–Gray sHR of 1.13, 95% CI 1.01–1.25; *p* = 0.031. The association was stronger and more consistent for type 2 endoleak, with a Cox HR of 1.26, 95% CI 1.12–1.43; *p* < 0.001, and a Fine–Gray sHR of 1.25, 95% CI 1.12–1.39; *p* < 0.001.

Age-stratified Fine–Gray subdistribution hazard ratios are reported in Supplementary Table S2.

### 3.5. Sensitivity Analyses and Propensity-Score Matching

Incremental model-fit analyses supported the added prognostic contribution of the baseline diameter beyond core anatomical features. Adding the baseline diameter improved the apparent Harrell’s C-index for any endoleak from 0.552 to 0.638 (ΔC-index, +0.085; ΔAIC, −29.6), for type 2 endoleak from 0.580 to 0.657 (ΔC-index, +0.077; ΔAIC, −25.4), and for reintervention from 0.579 to 0.628 (ΔC-index, +0.049; ΔAIC, −16.5). The improvement for all-cause death was modest, from 0.652 to 0.663 (ΔC-index, +0.011; ΔAIC, −10.2).

Device-adjusted models remained directionally concordant with the main analysis. In this device-available cohort, each 1 cm increase in the baseline diameter was associated with any endoleak (HR 1.35, 95% CI 1.19–1.52; *p* < 0.001), type 2 endoleak (HR 1.49, 95% CI 1.24–1.78; *p* < 0.001), reintervention (HR 1.19, 95% CI 1.04–1.37; *p* = 0.010), aneurysm-related mortality (HR 1.53, 95% CI 1.10–2.14; *p* = 0.012), and all-cause death (HR 1.08, 95% CI 1.01–1.16; *p* = 0.027).

In matched analyses across 50 imputed datasets, all 499 patients with aneurysms ≤50 mm were matched to 499 patients with aneurysms >50 mm in each imputation, with mean absolute standardized mean differences below 0.1 (Supplementary Figure S1).

In the anatomy-plus-age/sex-matched analysis, each 1 cm increase in the baseline diameter was associated with higher hazards of any endoleak (HR 1.25, 95% CI 1.14–1.38; *p* < 0.001), type 1a endoleak (HR 1.24, 95% CI 1.03–1.50; *p* = 0.023), type 2 endoleak (HR 1.26, 95% CI 1.13–1.40; *p* < 0.001), reintervention (HR 1.27, 95% CI 1.13–1.42; *p* < 0.001), and all-cause death (HR 1.16, 95% CI 1.05–1.27; *p* = 0.002). Center-adjusted, device-adjusted, and additional model-specification sensitivity analyses were directionally concordant for the main durability endpoints and are reported in Supplementary Table S3. Detailed and per-group results are reported in Table 5.

## 4. Discussion

In this large, multicenter, consecutive Italian EVAR cohort, the baseline aneurysm diameter was consistently associated with long-term EVAR durability across several analytical frameworks. The diameter used to inform treatment timing also appears to be associated with repair durability. Each additional centimeter of diameter was associated with approximately a 20% higher hazard of endoleak and reintervention. Our findings indicate that diameter contributes additional prognostic information beyond the classic anatomical descriptors, behaving as a distinct prognostic marker of EVAR durability [15].

These findings are prognostic and hypothesis-generating; they do not imply that earlier intervention in sub-threshold aneurysms would improve outcomes, as that question requires randomized evidence beyond the scope of this observational dataset.

Some endpoints required reduced models to avoid overfitting. Aneurysm-related death after EVAR was rare; when modeled with a limited covariate set (diameter, neck length, age), larger aneurysms showed higher hazards but with wide confidence intervals, a finding likely driven by the small number of events. These analyses highlight that rare endpoints should not be over-interpreted, but their directionality remained aligned with the overall pattern of size-dependent failure.

This approach is conservative in the present setting because several matched anatomical variables are tightly correlated with diameter and may lie on the causal pathway between aneurysm size and repair failure. The persistence of a diameter-associated gradient after matching therefore supports the robustness of the main finding, but it should not be interpreted as proof that diameter is fully separable from aneurysm morphology.

In Fine–Gray competing-risk models, the association between diameter and cumulative incidence was broadly consistent with Cox results, although competing mortality attenuated some age-stratified estimates.

These findings should be interpreted alongside, but not as a direct comparison with, UKSAT [5] and ADAM [7], which provide the randomized evidence underlying surveillance of small asymptomatic AAAs, showing no survival advantage for early open repair over ultrasound surveillance. Our findings do not challenge those data or current treatment thresholds. Instead, they address a post-repair question: among patients undergoing elective EVAR, the baseline aneurysm diameter remained associated with long-term durability, particularly endoleak and reintervention. This study cannot assess the appropriateness of treatment decisions. In such practice, repair at lower diameters may be driven by factors not captured by diameter alone, including rapid expansion, symptoms, concomitant iliac disease, patient preference, anatomical suitability, and center-specific multidisciplinary judgment.

Prior trials focused on rupture risk, whereas our data address EVAR durability across the size spectrum. Diameter behaved as a continuous risk factor for post-EVAR failure. Once EVAR is chosen, a larger sac at baseline heralds higher hazards of endoleak and reintervention and worse AAA-related survival. Although diameter may reflect underlying biomechanical factors that predispose to endograft failure, our study cannot establish causality; it only reports consistent associations across multiple analytic frameworks. Follow-up duration and surveillance intensity were not uniform across centers or across diameter strata; smaller aneurysms tended to undergo longer post-EVAR imaging follow-up by virtue of slower sac evolution and longer survival, which translates into more accumulated person–time at risk and may bias non-fatal event ascertainment in opposite directions for the two strata. Although competing-risk modeling addresses the survival-related bias, residual heterogeneity in surveillance intensity is a structural limitation of any retrospective multicenter real-world EVAR cohort and cannot be fully eliminated. Evidence on treatment outside device IFU aligns with our data [16,17,18]: outcomes are poorer out of IFU, and risk thresholds for type 1a failure tighten with short/large necks, which was also documented in Endurant series, including our own [18,19].

Device choice differed across diameter strata, with AFX used more frequently in smaller aneurysms and Endurant more frequently in larger aneurysms. Because some reports have associated AFX with late device-related complications, this imbalance would be expected to be biased against better durability in smaller aneurysms. Device-adjusted analyses remained directionally concordant with the primary models, supporting the interpretation that the observed diameter–durability gradient was not solely explained by graft selection.

The similar direction of results obtained with continuous diameter modeling and the a priori 50 mm stratification supports the presence of a diameter-related durability gradient. However, the 50 mm threshold should not be interpreted as an optimized predictive cut-off. Because the outcomes were time-to-event endpoints with variable follow-up and competing mortality, deriving a cut-off would risk oversimplifying an intrinsically time-dependent risk relationship. The clinical implication is therefore not to define a new treatment threshold, but to consider baseline sac size as one component of post-EVAR surveillance planning.

The associations reported in the present study do not imply that repairing smaller aneurysms leads to better outcomes; rather, they indicate that, among patients already selected for EVAR, the baseline diameter acts as a prognostic marker of post-procedural durability.

However, in the absence of a surveillance comparator, our data do not support lowering current size thresholds or advocating early EVAR for all small AAAs. Randomized or carefully matched comparative studies of early-elective EVAR versus continued surveillance could clarify whether intervening earlier confers a measurable advantage in long-term durability. Longitudinal imaging studies should further quantify neck progression in small versus larger aneurysms to understand how surveillance affects future EVAR feasibility and sealing length.

### Study Limitations

This study’s retrospective design introduces an inherent risk of selection bias. Symptom status and the individual indication for repair were not uniformly captured. Anatomical and procedural features were adjusted for multivariable models, but residual confounding, especially related to surgeon preference, device choice, and subtle neck characteristics not captured by routine measurements, cannot be excluded. Missing anatomical data were handled using multiple imputation, which reduces bias compared with complete-case analysis but relies on the assumption that data are missing at random. Fine–Gray models for rare endpoints (e.g., type 1a and 1b endoleaks, aneurysm-related death) may have been underpowered and susceptible to imprecision despite anatomically guided reduced models being employed to avoid overfitting.

Imaging follow-up intervals and adherence were not fully uniform across patients. The specific feeding vessel responsible for individual type 2 endoleaks was not formally recorded. Therefore, IMA-related and lumbar-related type 2 endoleaks could not be analyzed as separate endpoints. As an observational study among patients already selected for EVAR, the analysis cannot determine the absolute rupture risk prevented by repairing small aneurysms, nor can it estimate the comparative effectiveness of early EVAR versus continued surveillance.

## 5. Conclusions

Baseline aneurysm diameter was associated with EVAR durability in this multicenter cohort, with larger aneurysms associated with higher risks of endoleak, reintervention, and aneurysm-related mortality. Diameter provided added prognostic information beyond standard anatomical features, supporting its role in post-EVAR risk stratification.

The main clinical implication is for post-EVAR care, as baseline aneurysm diameter may help identify patients who warrant closer or more prolonged surveillance after repair, particularly when combined with anatomical, device-related, and early sac-behavior information. This study does not address the comparative effectiveness of early EVAR versus continued surveillance and should not be interpreted as evidence supporting earlier intervention.

## Figures and Tables

**Table 1:** Baseline and anatomical features.

Variable	Overall	AAA ≤ 50 mm	AAA > 50 mm	*p* Value
**Age (years)**	74 (68–80)	73 (68–78)	75 (69–81)	<0.001 *
**Female sex, n (%)**	124 (8.2%)	50 (10.0%)	74 (7.4%)	0.158
**Diabetes, n (%)**	290 (19.3%)	122 (24.4%)	168 (16.7%)	<0.001 *
**Arterial hypertension, n (%)**	1100 (78.9%)	360 (77.4%)	740 (79.6%)	0.391
**Dyslipidemia, n (%)**	750 (56.5%)	258 (61.1%)	492 (54.3%)	0.023 *
**Previous MI, n (%)**	250 (29.3%)	86 (27.2%)	164 (30.5%)	0.35
**COPD, n (%)**	726 (54.4%)	218 (50.7%)	508 (56.1%)	0.071
**CKD (GFR < 60) n (%)**	285 (21.9%)	82 (20.2%)	203 (22.6%)	0.37
**Proximal neck length (mm)**	20 (13–29)	23 (15–31.8)	20 (12–26.3)	<0.001 *
**Proximal neck diameter (mm)**	24 (22–26)	23 (21–25)	25 (22–27)	<0.001 *
**Proximal neck infrarenal angulation, °**	25 (14–42)	20 (8–29)	27 (15–44)	<0.001 *
**Right common iliac artery diameter (mm)**	14 (12–18)	14 (12–17)	15 (13–18)	<0.001 *
**Left common iliac artery diameter (mm)**	14 (12–18)	13.5 (11.4–16)	14.6 (12–18)	<0.001 *
**Patent lumbar arteries, median (IQR)**	3 (2–4)	4 (3–5)	3 (2–4)	<0.001 *

Continuous variables are presented as mean ± SD or median (IQR), and categorical variables are presented as counts and percentages. MI (myocardial infarction); COPD (chronic obstructive pulmonary disease); CKD (chronic kidney disease). Percentages are calculated among patients with available data. * Statistically significant.

**Table 2:** Early and follow-up outcomes. Variables are presented as counts and percentages.

Variable	Overall	AAA ≤ 50 mm	AAA > 50 mm	*p* Value
**In-hospital stay (days)**	4 (3–5)	4 (3–5)	4 (3–5)	0.282
**Oversizing (%)**	14 (6–21)	14 (6–23)	13 (5–22)	0.348
**Follow-up time (months)**	49 (20–78)	55 (23–84)	43 (16–74)	<0.001 *
**Any endoleak n (%)**	290 (19.3)	55 (11.1)	235 (23.7)	<0.001 *
**Type 1a**	86 (5.7)	14 (2.8)	72 (7.2)	<0.001 *
**Type 1b**	34 (2.3)	7 (1.4)	27 (2.7)	0.117
**Type 2**	184 (12.2)	35 (7.0)	149 (14.8)	<0.001 *
**Type 3**	26 (1.7)	11 (2.2)	15 (1.5)	0.317
**Branch thrombosis n (%)**	28 (1.9)	4 (0.8)	24 (2.4)	0.032*
**Reintervention n (%)**	188 (12.5)	34 (6.8)	154 (15.3)	<0.001 *
**All-cause death n (%)**	577 (38.3)	137 (27.5)	440 (43.7)	<0.001 *
**Aneurysm-related death n (%)**	25 (1.7)	3 (0.6)	22 (2.2)	0.030 *
**Sac variation at last follow-up**				
>5 mm shrink	516 (59.7)	166 (62.6)	350 (58.4)	0.245
Stable	201 (23.7)	73 (27.8)	128 (21.7)	0.044 *
Increase > 5 mm	147 (17.0)	25 (9.3)	122 (20.1)	<0.001 *

* Statistically significant.

**Table 3:** Cumulative incidence functions and Gray’s test (death = competing risk).

Outcome	Group	Events	Competing Events	3-Year CIF	5-Year CIF	10-Year CIF	Gray *p*
**Any endoleak**	≤50 mm	55	109	8.1%	11.1%	15.8%	<0.001 *
**Any endoleak**	>50 mm	235	343	17.3%	19.6%	33.9%	
**Type 1a endoleak**	≤50 mm	14	125	1.0%	2.2%	6.1%	0.001 *
**Type 1a endoleak**	>50 mm	72	403	3.2%	4.0%	16.5%	
**Type 1b endoleak**	≤50 mm	7	129	0.4%	1.3%	2.2%	0.130
**Type 1b endoleak**	>50 mm	27	419	1.7%	2.1%	3.9%	
**Type 2 endoleak**	≤50 mm	35	118	6.7%	7.0%	7.9%	<0.001 *
**Type 2 endoleak**	>50 mm	149	382	12.6%	13.3%	18.2%	
**Reintervention**	≤50 mm	34	118	2.7%	4.4%	16.2%	<0.001 *
**Reintervention**	>50 mm	154	377	7.5%	11.2%	24.7%	
**Aneurysm-related mortality**	≤50 mm	3	129	0.3%	0.3%	2.4%	0.028 *
**Aneurysm-related mortality**	>50 mm	22	413	0.5%	1.2%	5.4%	

* Statistically significant.

**Table 4:** Full/core anatomical models included age, sex, proximal neck length, proximal neck diameter, iliac diameters, and patent lumbar arteries. Reduced anatomical models included age, sex, proximal neck length, and proximal neck diameter and were used for type-specific or rare endpoints to avoid overfitting.

Outcome	Model Used for Main Estimate	Cox HR per cm	*p*	Fine–Gray sHR per cm	*p*
Any endoleak	Full/core anatomical	1.22 (1.11–1.34)	<0.001	1.20 (1.10–1.31)	<0.001 *
Type 1a endoleak	Reduced anatomical	1.21 (1.06–1.38)	0.006	1.13 (1.01–1.25)	0.031
Type 1b endoleak	Reduced anatomical	1.17 (0.92–1.48)	0.205	1.10 (0.87–1.38)	0.443
Type 2 endoleak	Full/core anatomical	1.26 (1.12–1.43)	<0.001	1.25 (1.12–1.39)	<0.001 *
Reintervention	Full/core anatomical	1.19 (1.07–1.32)	0.001	1.15 (1.05–1.27)	0.004 *
Aneurysm-related mortality	Reduced anatomical	1.46 (1.19–1.81)	<0.001	1.40 (1.14–1.71)	0.001 *
All-cause death	Full/core anatomical	1.09 (1.02–1.17)	0.016	Not applicable	—

* Statistically significant.

**Table 5:** Cox proportional hazards model for all outcomes (imputed matched dataset). Values are hazard ratios (HR) with 95% confidence intervals (CI).

Outcome	HR Per cm in Matched Cohort	*p*	HR for ≤50 mm vs. >50 mm	*p*
Any endoleak	1.25 (1.14–1.38)	<0.001 *	0.39 (0.28–0.55)	<0.001 *
Type 1a endoleak	1.24 (1.03–1.50)	0.023 *	0.44 (0.22–0.90)	0.025 *
Type 1b endoleak	1.15 (0.79–1.66)	0.465	0.58 (0.21–1.59)	0.287
Type 2 endoleak	1.26 (1.13–1.40)	<0.001 *	0.38 (0.25–0.58)	<0.001 *
Reintervention	1.27 (1.13–1.42)	<0.001 *	0.40 (0.26–0.62)	<0.001 *
Aneurysm-related mortality	1.34 (0.85–2.12)	0.206	0.34 (0.08–1.49)	0.153
All-cause death	1.16 (1.05–1.27)	0.002*	0.64 (0.50–0.82)	<0.001 *

* Statistically significant (*p* < 0.05).

## Data Availability

The original contributions presented in this study are included in the article. Further inquiries can be directed to the corresponding author.

## Supplementary Materials

The following supporting information can be downloaded at: https://www.mdpi.com/article/10.3390/jcdd13070325/s1. Supplementary Table S1: Missingness and analytic handling of key variables; Supplementary Table S2: Age-stratified Fine–Gray subdistribution hazard ratios (per cm of baseline diameter; death as competing event); Supplementary Table S3: Center-adjusted sensitivity analyses; Supplementary Figure S1: Love plot showing standardized mean difference (SMD) across covariates before and after matching.

## Abbreviations

The following abbreviations are used in this manuscript:

AAA	Abdominal aortic aneurysm
ADAM	Aneurysm Detection and Management study
AFX	AFX endograft system
AIC	Akaike information criterion
CI	Confidence interval
CIF	Cumulative incidence function
CIFL	Interuniversity Center of Phlebolymphology
CKD	Chronic kidney disease
COPD	Chronic obstructive pulmonary disease
CTA	Computed tomography angiography
ESVS	European Society for Vascular Surgery
EVAR	Endovascular aneurysm repair
GFR	Glomerular filtration rate
HR	Hazard ratio
IFU	Instructions for use
IQR	Interquartile range
IRCCS	Istituto di Ricovero e Cura a Carattere Scientifico
JASP	JASP statistical software version 0.97.0
LLC	Limited liability company
MI	Myocardial infarction
ORCID	Open Researcher and Contributor ID
SD	Standard deviation
sHR	Subdistribution hazard ratio
SICVE	Italian Society of Vascular and Endovascular Surgery
STROBE	Strengthening the Reporting of Observational Studies in Epidemiology
UKSAT	United Kingdom Small Aneurysm Trial
USA	United States of America
VQI	Vascular Quality Initiative
