# Supplementary material for: Preoperative Abdominal Aortic Aneurysm Diameter Is Associated with Long-Term Durability After Endovascular Aortic Aneurysm Repair: A Multicenter Real-World Italian Cohort Study
Source: J Cardiovasc Dev Dis. 2026 Jul 12;13(7):325. doi: 10.3390/jcdd13070325 (PMC13409815; doi:10.3390/jcdd13070325)

Supplementary Figure 1. Love plot showing standardized mean difference (SMD) across covariates before and after matching

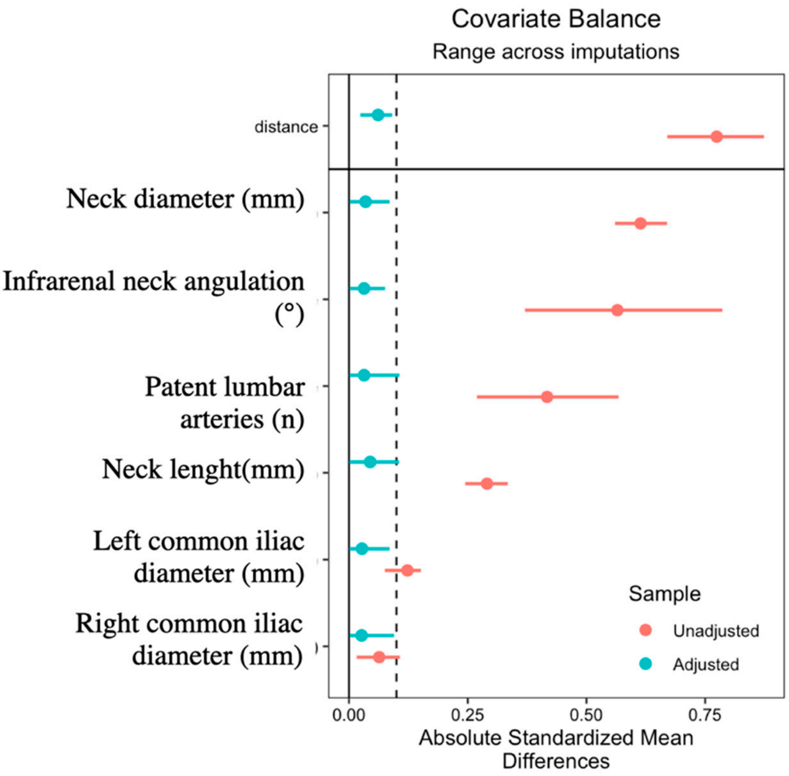

Supplement: Supplementary file 1 [file jcdd-13-00325-s001.zip › Supplementary Figure S1.pdf]
