# Supplementary material for: Preoperative Abdominal Aortic Aneurysm Diameter Is Associated with Long-Term Durability After Endovascular Aortic Aneurysm Repair: A Multicenter Real-World Italian Cohort Study
Source: J Cardiovasc Dev Dis. 2026 Jul 12;13(7):325. doi: 10.3390/jcdd13070325 (PMC13409815; doi:10.3390/jcdd13070325)
Supplement: Supplementary file 1 [file jcdd-13-00325-s001.zip › Supplementary Table S1.pdf]

Supplementary Table S1. Missingness and analytic handling of key variables

Variable	Available, n/N	Missing, n (%)	Analytic handling
Baseline aneurysm diameter	1505/1505	0 (0.0%)	Primary exposure; not imputed
Age	1449/1505	56 (3.7%)	Included in imputation/modeling
Sex	1505/1505	0 (0.0%)	Not imputed
Proximal neck length	1306/1505	199 (13.2%)	Imputed for multivariable/matched analyses
Proximal neck diameter	1397/1505	108 (7.2%)	Imputed for multivariable/matched analyses
Infrarenal neck angulation	976/1505	529 (35.1%)	Categorical descriptor/sensitivity variable
Right common iliac artery diameter	1371/1505	134 (8.9%)	Imputed for multivariable/matched analyses
Left common iliac artery diameter	1364/1505	141 (9.4%)	Imputed for multivariable/matched analyses
Patent lumbar arteries	796/1505	709 (47.1%)	Imputed for multivariable/matched analyses
Oversizing	1058/1505	447 (29.7%)	Descriptive/sensitivity variable
Device type	1071/1505	434 (28.8%)	Not imputed; device-adjusted analyses restricted to available cases
Sac behavior at last follow-up	864/1505	661 (43.9%)	Not imputed; descriptive available-case analysis
