# Supplementary material for: Preoperative Abdominal Aortic Aneurysm Diameter Is Associated with Long-Term Durability After Endovascular Aortic Aneurysm Repair: A Multicenter Real-World Italian Cohort Study
Source: J Cardiovasc Dev Dis. 2026 Jul 12;13(7):325. doi: 10.3390/jcdd13070325 (PMC13409815; doi:10.3390/jcdd13070325)
Supplement: Supplementary file 1 [file jcdd-13-00325-s001.zip › Supplementary Table S2.pdf]

Supplementary Table S2. Age-stratified Fine–Gray subdistribution hazard ratios (per cm of baseline diameter; death as competing event)

Pooled across 50 imputed datasets using Rubin's rules (manual implementation; cmprsk::crr fitted within each dataset, coefficients pooled).

Outcome	<70 yr (n 398)	70–79 yr (n 634)	≥80 yr (n 417)
Any endoleak	1.09 (0.96–1.24) p=0.19	**1.23 (1.13–1.35) p<0.001**	1.07 (0.93–1.22) p=0.36
Type 1a endoleak	1.11 (0.92–1.35) p=0.28	**1.35 (1.20–1.53) p<0.001**	1.02 (0.81–1.27) p=0.89
Type 1b endoleak	**1.30 (1.05–1.61) p=0.018**	1.18 (0.86–1.62) p=0.31	1.13 (0.78–1.65) p=0.51
Type 2 endoleak	1.03 (0.84–1.24) p=0.80	**1.22 (1.09–1.36) p<0.001**	1.07 (0.92–1.24) p=0.41
Reintervention	1.09 (0.96–1.24) p=0.18	**1.26 (1.13–1.39) p<0.001**	1.15 (0.98–1.35) p=0.082
Aneurysm-related death	0.99 (0.56–1.77) p=0.98	**1.53 (1.06–2.21) p=0.023**	**1.62 (1.24–2.12) p<0.001**

Exploratory interpretation: Age-stratified analyses suggested that the diameter-associated gradient for non-fatal durability endpoints was mostly present in patients aged 70–79 years. In patients aged ≥80 years, competing non-aneurysm mortality attenuated non-fatal endpoint estimates, whereas aneurysm-related mortality remained associated with baseline diameter. These findings should be considered hypothesis-generating.
