# Supplementary material for: Preoperative Abdominal Aortic Aneurysm Diameter Is Associated with Long-Term Durability After Endovascular Aortic Aneurysm Repair: A Multicenter Real-World Italian Cohort Study
Source: J Cardiovasc Dev Dis. 2026 Jul 12;13(7):325. doi: 10.3390/jcdd13070325 (PMC13409815; doi:10.3390/jcdd13070325)
Supplement: Supplementary file 1 [file jcdd-13-00325-s001.zip › Supplementary Table S3.pdf]

Supplementary Table S3. Center-adjusted sensitivity analyses.

Outcome	Center-stratified Cox HR per cm	Cox n/events	Center-adjusted Fine–Gray sHR per cm	Fine–Gray n/events
Any endoleak	1.23 (1.12–1.35); p<0.001	1504/290	1.21 (1.11–1.32); p<0.001	1504/290
Type Ia endoleak	1.10 (0.94–1.29); p=0.242	1504/86	1.05 (0.92–1.19); p=0.504	1504/86
Type Ib endoleak	1.14 (0.90–1.45); p=0.268	1504/34	1.07 (0.85–1.35); p=0.585	1504/34
Type II endoleak	1.30 (1.15–1.48); p<0.001	1504/183	1.29 (1.16–1.44); p<0.001	1504/183
Reintervention	1.19 (1.07–1.33); p=0.001	1504/187	1.15 (1.05–1.27); p=0.004	1504/187
Aneurysm-related mortality	1.53 (1.23–1.91); p<0.001	1505/25	1.47 (1.18–1.83); p<0.001	1505/25
**Outcome**	**Device-adjusted Cox HR per cm**	**n/events**	—	—
Any endoleak	1.35 (1.19–1.52); p<0.001	1070/126		
Type Ia endoleak	1.13 (0.92–1.40); p=0.243	1070/44		
Type Ib endoleak	1.24 (0.97–1.57); p=0.082	1070/28		
Type II endoleak	1.49 (1.24–1.78); p<0.001	1070/55		
Reintervention	1.19 (1.04–1.37); p=0.010	1070/100		
Aneurysm-related mortality	1.53 (1.10–2.14); p=0.012	1071/10		
All-cause death	1.08 (1.01–1.16); p=0.027	1070/434		

Note. Values are hazard ratios or subdistribution hazard ratios per 1-cm increase in baseline aneurysm diameter. Center-stratified Cox models allowed center-specific baseline hazards; center-adjusted Fine–Gray models included center terms and center-specific censoring groups.

Device-adjusted models were restricted to patients with available device information. Device group was categorized as Endurant, AFX, or other.
